# Supplementary material for: Reconciling Mining with the Conservation of Cave Biodiversity: A Quantitative Baseline to Help Establish Conservation Priorities
Source: PLoS One. 2016 Dec 20;11(12):e0168348. doi: 10.1371/journal.pone.0168348 (PMC5173368; doi:10.1371/journal.pone.0168348)
Supplement: S1 Dataset — (ZIP) [file pone.0168348.s002.zip › Taxa/Serra Norte/SN_2013/N4WS/Lista N4WS-27.pdf]

CAVIDADE N4WS-0027

Classe	Ordem	Fam/Outros	Gên/Outros	Espécie	S-U	U-S	Zon.
Annelida	Oligochaeta			sp.	1	1	E/P
Arachnida	Acari	Lohmanniidae		sp.1	X		P
Arachnida	Acari	Rhagidiidae		sp.1	X		E
Arachnida	Acari	Veigaiidae	*Veigaia*	sp.1	X		E
Arachnida	Amblypygi	Phrynidae	*Heterophrynus*	*longicornis*	4	2	E/P
Arachnida	Araneae	Oonopidae		jovem		X	P
Arachnida	Araneae	Pholcidae	*Mesabolivar*	*aurantiacus*	X	X	P
Arachnida	Araneae	Tetragnathidae		jovem		X	
Arachnida	Araneae	Theridiosomatidae	*Plato*	sp.1	X	X	P
Arachnida	Araneae	Trechaleidae	*Rhoicinus*	sp.1		2	
Arachnida	Opiliones	Cosmetidae		sp.2	1		E
Arachnida	Opiliones	Sclerosomatidae	*Prionostema*	sp.1		X	E
Arachnida	Pseudoscorpiones	Chernetidae	*Spelaeochernes*	sp.1	X	X	P
Arachnida	Pseudoscorpiones	Chthoniidae	*Pseudochthonius*	sp.1	X	X	P
Chilopoda	Geophilomorpha	Geophilidae		sp.2	1		E
Diplopoda	Polydesmida			sp.	X		P
Entognatha	Collembola	Isotomidae		sp.1	X	X	P
Entognatha	Collembola	Paronellidae		sp.1		X	P
Entognatha	Diplura	Campodeidae		sp.1		X	P
Insecta	Coleoptera	Carabidae		jovem		X	P
Insecta	Coleoptera	Leiodidae		sp.4	X		P
Insecta	Coleoptera	Staphylinidae	Pselaphinae	sp.14	X		E
Insecta	Coleoptera	Staphylinidae	Pselaphinae	sp.40	X		P
Insecta	Coleoptera	Staphylinidae	Pselaphinae	sp.9		X	P
Insecta	Diptera	Cecidomyiidae	Cecidomyiinae	sp.	X		P
Insecta	Diptera	Ceratopogonidae		jovem	X		P
Insecta	Diptera	Phoridae	Metopininae	sp.	X		P
Insecta	Diptera	Psychodidae	*Sciopemyia*	*sordellii*	X		E
Insecta	Diptera	Tipulidae	Tipulinae	sp.	X		P
Insecta	Heteroptera	Cydnidae	Cydninae	jovem		X	P
Insecta	Heteroptera	Veliidae	*Paravelia*	sp.1	X		E/P
Insecta	Hymenoptera	Formicidae	*Crematogaster*	sp.3		X	P
Insecta	Hymenoptera	Formicidae	*Dolichoderus*	aff. *bispinosus*		X	E
Insecta	Hymenoptera	Formicidae	*Pachycondyla*	*striata*	X		P
Insecta	Hymenoptera	Formicidae	*Rogeria*	sp.1	X		P
Insecta	Hymenoptera	Formicidae	*Solenopsis*	sp.1	X		E
Insecta	Hymenoptera	Formicidae	*Trachymyrmex*	sp.2		X	P
Insecta	Isoptera	Termitidae	*Nasutitermes*	sp.	X		P
Insecta	Lepidoptera	Noctuoidea		sp.2	X		P
Insecta	Orthoptera	Phalangopsidae	*Paraclodes*	sp.	3		E/P
Insecta	Orthoptera	Phalangopsidae	*Phalangopsis*	sp.	8	34	P
Insecta	Psocoptera	Epipsocidae	*Epipsocus*	sp.2	X		E
Insecta	Psocoptera	Psyllipsocidae	*Psyllipsocus*	sp.3		X	
Gastropoda	Pulmonata	Subulinidae	*Lamellaxis*	sp.		X	P
Malacostraca	Isopoda	Philosciidae		sp.2	X		E
Symphyla		Scutigerellidae	*Hanseniella*	sp.1		X	P
Amphibia	Anura	Strabomantidae	*Pristimantis*	cf. *fenestratus*	1	2	E/P
